# Low‐Dimensional Metal Halide Perovskite Crystal Materials: Structure Strategies and Luminescence Applications

**DOI:** 10.1002/advs.202004805

**Published:** 2021-06-17

**Authors:** Ying Han, Sijia Yue, Bin‐Bin Cui

**Affiliations:** ^1^ Advanced Research Institute of Multidisciplinary Science Beijing Institute of Technology (BIT) Beijing 100081 P. R. China; ^2^ Department of Materials Science and Engineering BIT Beijing 100081 P. R. China; ^3^ School of Materials Science and Engineering BIT Beijing 100081 P. R. China

**Keywords:** light emitting diodes, low‐dimensional perovskites, luminescence, phosphors, structure regulation

## Abstract

Replacing methylammonium (MA^+^), formamidine (FA^+^), and/or cesium (Cs^+^) in 3D metal halide perovskites by larger organic cations have built a series of low‐dimensional metal halide perovskites (LDMHPs) in which the inorganic metal halide octahedra arranging in the forms of 2D layers, 1D chains, and 0D points. These LDMHPs exhibit significantly different optoelectronic properties from 3D metal halide perovskites (MHPs) due to their unique quantum confinement effects and large exciton binding energies. In particular, LDMHPs often have excellent broadband luminescence from self‐trapped excitons. Chemical composition, hydrogen bonding, and external factors (temperature and pressure etc.) determine structures and influence photoelectric properties of LDMHPs greatly, and especially it seems that there is no definite regulation to predict the structure and photoelectric properties when a random cation, metal, and halide is chosen to design a LDMHP. Therefore, this review discusses the construction strategies of the recent reported LDMHPs and their application progress in the luminescence field for a better understanding of these factors and a prospect for LDMHPs’ development in the future.

## Graphical abstract

Chemical composition, hydrogen bonding, and external factors (temperature and pressure) determine the structures and photoelectric properties of low‐dimensional metal halide perovskites (LDMHPs). This review discusses the construction strategies of the recent LDMHPs and their applications in the luminescence field for a better understanding of these factors and a prospect for LDMHPs’ development in the future.

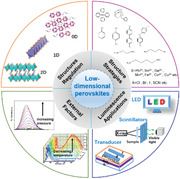

## 1 Introduction

As promising photoelectric materials, metal halide perovskites (MHPs) have been widely studied and applied to photovoltaic devices, photodetectors, light emitting diodes (LEDs), etc.^[^ ^1^ ^]^ In particular, MHPs have unique structure tunability, the FA^+^, MA^+^, or Cs^+^ in 3D MHPs replaced by larger sized organic cations can build various low‐dimensional MHPs (LDMHPs) at the molecular level, such as 2D, 1D, and 0D metal halide crystal materials.^[^ ^2^ ^]^ 3D MHPs (ABX_3,_ A stands for monovalent cation, such as FA^+^, MA^+^, Cs^+^; M represents metal ions, such as Pb^2+^, Sn^2+^, Ge^2+^ etc., and X stands for halogen ion Cl^−^, Br^−^, I^−^)^[^ ^3^ ^]^ with the advantages of high absorption coefficient, adjustable optical bandgap, low exciton binding energy, and long carrier diffusion distance have been widely used in the field of solar cells with a certified power conversion efficiency (PCE) of world recorded over 25%.^[^ ^4^ ^]^ In general, 3D perovskites have small exciton binding energies (≈20–50 meV),^[^ ^5^ ^]^ which lead to their low radiation recombination efficiency and photoluminescent quantum efficiency (PLQE), and limit their application in luminescence.^[^ ^6^ ^]^ On the contrary, LDMHPs commonly exhibit excellent luminescence due to their quantum confined effect and large exciton binding energies.^[^ ^7^ ^]^ Different from nanosheets, nanowires, and quantum dots at the structural level, LDMHPs discussed in this work refer to the low‐dimensional crystals at the molecular level in particular.

The selection of cations, metals, and halogens play a key role in determining chemical composition and crystal structures of LDMHPs. Crystals with different dimensions were constructed by selecting the size of organic cations, and regulating metal cations could also construct LDMHPs with different dimensions.^[^ ^8^ ^]^ In addition to single metal, double perovskite, and quadruple perovskites could be formed by mixing metal cations.^[^ ^9^ ^]^ The change of halogen could regulate the configuration and the distortion degree of LDMHPs, which is the key to realize the broadband white luminescence.^[^ ^10^ ^]^ Generally, controlling growth of all‐inorganic Cs‐Pb‐X perovskites with different dimensions could be easily realized by regulating the stoichiometric ratio of the precursor solution during the crystal growth progress.^[^ ^11^ ^]^ However, for organic–inorganic hybrid perovskites, a kind organic cation usually only constructed a structure even the stoichiometric ratio of every elements in the precursor solution was changed unless it formed different valence states or interacted with other molecules. On the other hand, manipulating the hydrogen bonding interactions between the inorganic skeletons and organic cations could significantly affect the orientation and conformation of inorganic skeleton.^[^ ^12^ ^]^ Furthermore, external pressure and temperature will provide thermodynamic energies to change the structures and photoelectric properties of LDMHPs.^[^ ^13^, ^14^ ^]^

Recently, it has spawned the “diamond fever” on the family of LDMHPs, and they have been intensely studied and applied as promising new photoelectric functional materials due to excellent luminescence properties and good stabilities.^[^ ^15^ ^]^ In particular, LDMHPs exhibit typical luminescent properties of large stokes shift and broadband emission,^[^ ^16^ ^]^ which could achieve tunable luminescence covering the whole visible light region and single phase white light emission.^[^ ^17^ ^]^ Compared to 3D perovskites, the summarization on the structural design strategy and its relationship with photoelectric properties of LDMHPs are still very rare. In this article, the specific discussion on the construction strategies related to alteration of chemical constituents including cations, metal ions, halide anions, stoichiometric ratio, hydrogen bonding, temperature, and pressure will be mainly reviewed. In addition, LDMHPs’ broadband emissions and their application in LED devices and phosphors in solid lighting are discussed here to have a systematic and prospective knowledge of promotion in luminescence properties when different structures of LDMHPs are selected.

## 2 Structure Construction and Luminescence Performance

### 2.1 Cations

**Figure 1:**
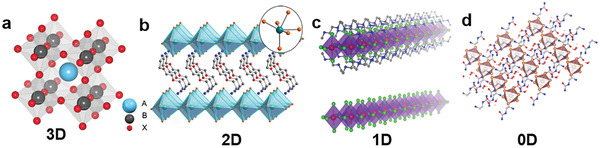
a) Typical single crystal structures of 0D–3D organic–inorganic hybrid perovskites. 3D. Reproduced with permission.^[^ ^15^ ^]^ Copyright 2018, Wiley‐VCH. b) 2D. Reproduced with permission.^[^ ^17^ ^]^ Copyright 2014, American Chemical Society. c) 1D. Reproduced with permission.^[^ ^19^ ^]^ Copyright 2017, Springer Nature. d) 0D. Reproduced with permission.^[^ ^12^ ^]^ Copyright 2019, Springer Nature.

As shown in **Figure** **1**a, the common structure of the 3D perovskite is AMX_3_, in which the metal atoms are in the center and the halogen atoms are in the vertexes of the unit octahedrons extending in a 3D mode. The organic or inorganic monovalent cations are located in the cavities constructed by [MX_6_^4−^] octahedrons, which is conforming to the theoretical formula of $RA+RX=t\sqrt{2}\left(RM+RX\right)$, where *R*_A_, *R*_M_, and *R*_X_ represent the radius of the A, M, X ions and *t* is the tolerance factor, respectively. For example, result from the construction formula limit, when *t* = 1, *R*_M_ and *R*_X_ are the maximum values (*R*_Pb_ = 1.19 Å, *R*_I_ = 2.20 Å), the maximum *R*_A_ approximately is 2.6 Å. Therefore, only small cations which maximum lengths are shorter than 2.6 Å can satisfy the condition to build 3D APbI_3_ perovskites.^[^ ^18^ ^]^ Correspondingly, replacing the FA^+^, MA^+^, or Cs^+^ with larger size organic cations, abundant LDMHPs can be obtained, including layered 2D, linear 1D and points distributed 0D metal halide hybrids (Figure 1b–d).

**Figure 2:**
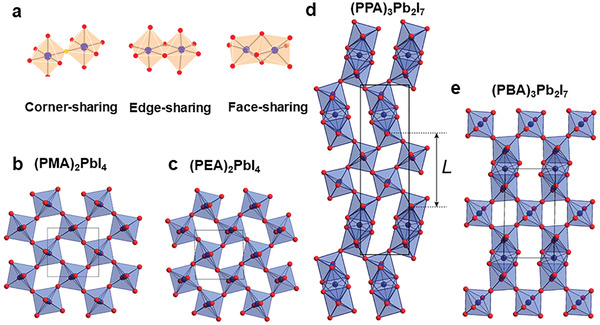
a) 2D structure of corner‐sharing, edge‐sharing and face‐sharing. Reproduced with permission.^[^ ^7^ ^]^ Copyright 2019, American Chemical Society. b–e) 2D perovskites constructed by PMA, PEA, PPA, and PBA, respectively. Reproduced with permission.^[^ ^20^ ^]^ Copyright 2016, American Chemical Society.

The size of cations affects the connection modes of lead halide octahedrons. There are three types of connection modes for metal halide perovskites: corner‐sharing, edge‐sharing, and face‐sharing as shown in **Figure** **2**a. Although most configurations of 2D perovskites are corner‐sharing, a few 2D perovskites have broken the conventional connection mode and formed edge‐sharing and face‐sharing structures. The types of connection modes can be regulated by changing the length of amines.^[^ ^7^ ^]^ For example, as shown in Figure 2b–e, C_6_H_5_(CH_2_)*_n_* NH_3_^+^, where *n* = 1, 2, 3, 4 represents organic phenylammonium (PMA), phenylethylammonium (PEA), phenylethylammonium (PPA), and phenylethylammonium (PBA), respectively. The 2D structures constructed by PMA and PEA are corner‐sharing, while the 2D structures constructed by the longer PPA and PBA are corner‐sharing and face‐sharing coexistence.^[^ ^20^ ^]^ Recently, the post‐perovskite with edge‐sharing structure formed by organic cation *trans*‐2, 5‐dimethylpiperazine has been reported. Due to the distorted inorganic lead halogen structure, it exhibits white emission with a high PLQE.^[^ ^21^ ^]^ Furthermore, the connection mode influences the orbital overlap between metals and halogens, thus affecting the bandgap values of 2D perovskites, where it is common that face‐sharing>edge‐sharing>corner‐sharing for bandgap values.^[^ ^7^ ^]^

For common 2D perovskites, organic cations which are suitable for 2D interlayer frames often contain a positive charge (e.g., RNH_3_^+^, R_2_NH_2_^+^, R_3_NH^+^, R_4_N^+^ (R is a paraffinic chain)); hydrogen atoms of the organic amine can interact with halide atoms on metal halide octahedrons by hydrogen bonding. The distance between the inorganic layers in 2D perovskites can be adjusted by organic cations with short or long paraffinic chains, and perovskites in lower dimensions can be obtained by adjusting longer paraffinic chains. For example, 2D perovskites (R*_n_* NH_3_)_2_MX_4_ and (NH_3_R*_n_* NH_3_)MX_4_ constructed by monoamine and diamine organic cations are shown in Figure 4. For (R*_n_* NH_3_)_2_MX_4_, when *n* = 1, methylamine cation construct the common 3D perovskite MAPbX_3_,^[^ ^22^ ^]^ and when *n* = 2, ethylamine, it construct the 2D perovskites EA_4_Pb_3_X_10_.^[^ ^10^ ^]^ EA has two configurations in two positions, as some of the cations are filled into 3D perovskite cavity and another part of the EA that splits into inorganic layer to form a 2D perovskite. In addition, when *n* > 2, the organic cations will construct single layer 2D perovskites.^[^ ^23^ ^]^ For (NH_3_R*_n_* NH_3_)MX_4_, the diamine organic cations are long enough to link the two inorganic layers as the n values increases. For example, when *n* ≥ 4, such as [NH_3_(CH_2_)*_n_* NH_3_]PbI_4_ (*n* = 4, 6, 8), the diamine organic cations are long enough to construct regular 100‐oriented 2D perovskites.^[^ ^24^ ^]^ The other good example is that, for butyric diamine (*n* = 4), propylene diamine (*n* = 3), and ethylenediamine (*n* = 2), as the length of the amine chain decreases, the layered perovskite changes from a 100‐oriented flat layer to a distorted layer, and then when *n* = 2, the layer broken to construct a 1D ribbon structure.^[^ ^25^ ^]^

**Figure 3:**
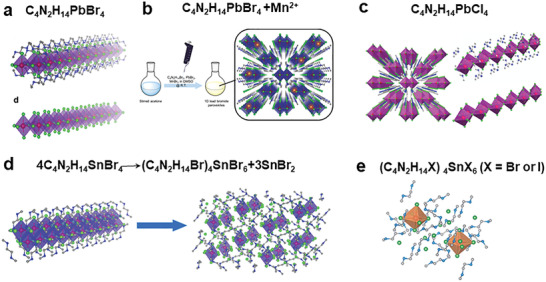
a) Structure of 1D organic lead halide perovskites C_4_N_2_H_14_PbBr_4_ constructed by C_4_N_2_H_14_^2+^. Reproduced with permission.^[^ ^19^ ^]^ Copyright 2017, Springer Nature. b) Preparation of 28% white light emission system with 1D C_4_N_2_H_14_PbBr_4_ doping Mn^2+^. Reproduced with permission.^[^ ^26^ ^]^ Copyright 2017, American Chemical Society c) Structure of 1D organic lead halide perovskites C_4_N_2_H_14_PbCl_4_. Reproduced with permission.^[^ ^27^ ^]^ Copyright 2018, American Chemical Society. d) 1D Sn‐based C_4_N_2_H_14_SnBr_6_ transformed into a 0D structure (C_4_N_2_H_14_Br)_4_SnBr_6_. Reproduced with permission.^[^ ^28^ ^]^ Copyright 2017, Wiley‐VCH. e) Structure of core–shell 0D perovskites (C_4_N_2_H_14_X)_4_S*_n_* X_6_ (X = Br, I). Reproduced with permission.^[^ ^29^ ^]^ Copyright 2018, RoyalSociety of Chemistry.

**Figure 4:**
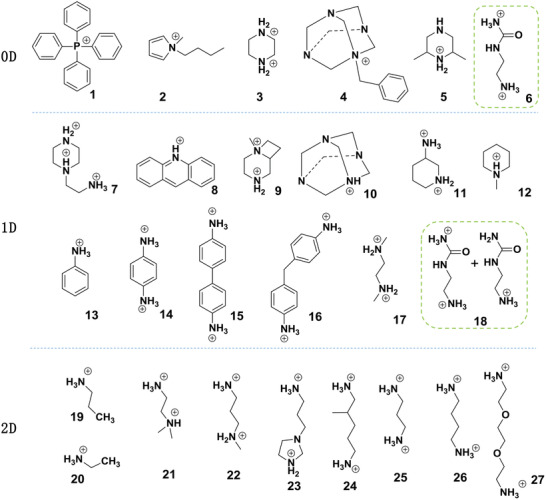
The reported organic molecules with large cross‐sectional area for constructing 1D and 0D perovskites (Ref:1,^[^ ^30^ ^]^ 2,^[^ ^29^ ^]^ 3,^[^ ^33^ ^]^ 4,^[^ ^34^ ^]^ 5,^[^ ^35^ ^]^ 6,^[^ ^12^ ^]^ 7,^[^ ^36^ ^]^ 8,^[^ ^37^ ^]^ 9,^[^ ^38^ ^]^ 10,^[^ ^39^ ^]^ 11,^[^ ^40^ ^]^ 12,^[^ ^41^ ^]^ 13,^[^ ^42^ ^]^ 14,^[^ ^43^ ^]^ 15,^[^ ^44^ ^]^ 16,^[^ ^24^ ^]^ 17^[^ ^29^ ^]^, 18,^[^ ^12^ ^]^ 19,^[^ ^23^ ^]^ 20, ^[^ ^10^ ^]^ 21^[^ ^45^ ^]^, 22,^[^ ^46^ ^]^ 23,^[^ ^47^ ^]^ 24,^[^ ^48^ ^]^ 25,^[^ ^25^ ^]^ 26,^[^ ^25^ ^]^ 27^[^ ^17^ ^]^).

Recently, Ma's group synthesized multiple perovskites with 1D and 0D structures by diamine cation with the *N, N*‐2‐dimethylethylenediamine (organic cation 17 in Figure 4). Such as the 1D C_4_N_2_H_14_PbBr_4_ with efficient bluish white‐light emission,^[^ ^19^ ^]^ as shown in **Figure** **3**a. The C_4_N_2_H_14_PbBr_4_ achieved white light emission with PLQE of 28% by doping Mn^2+^ (Figure 3b).^[^ ^26^ ^]^ The author also reported 1D perovskite C_4_N_2_H_14_PbCl_4_ (Figure 3c) and studied its structure distortion.^[^ ^27^ ^]^ As shown in Figure 3d, from no luminescence property to yellowish‐white emission, 1D Sn‐based C_4_N_2_H_14_SnBr_6_ is easier to transform into a 0D of (C_4_N_2_H_14_Br)_4_SnBr_6_.^[^ ^28^ ^]^ And the Sn‐based 0D (C_4_N_2_H_14_X)_4_SnX_6_ (X = Br^−^, I^−^) has a PLQE of nearly unity (Figure 3e).^[^ ^29^ ^]^

Although the organic cations in 2D structure are not as small as the cations in 3D perovskites, they still need to fit into the frame of 2D inorganic layers, and the cross‐sectional area of organic molecule must be suitable to the square range constructed by the four corner‐sharing lead halide octahedrons, allowing the organic molecule to tilt and interlace, where the side length of the square is greater than or equal to twice the average bond length of the Pb‐X. The space cannot accommodate the too large adjacent organic molecules, because the larger cations more possibly construct lower dimensional structures, such as 1D and 0D structures. For organic molecules with large cross‐sectional area, it's more likely to break up the 2D inorganic layers to form a 1D or 0D structure. For example, the large circular organic molecules C_9_NH_20_^+^ (organic cation 2 in **Figure** **4**) constructed a variety of 0D MHPs, such as (C_9_NH_20_)_7_(PbCl_4_)Pb_3_Cl_11_ with a blue emission,^[^ ^31^ ^]^ (C_9_NH_20_)_2_SnBr_4_ with a deep‐red emission,^[^ ^32^ ^]^(C_9_NH_20_)_2_SbCl_5_ with a yellow emission,^[^ ^29^ ^]^ and (bmpy)_9_[ZnCl_4_]_2_[Pb_3_Cl_11_]^[^ ^12^ ^]^ with a green emission, etc. Organic cations with large cross‐sectional area in 1D and 0D structures are summarized in Figure 4, and the optical properties of these LDMHPs constructed by corresponding molecules are shown in **Table** **1**. In summary, the size of the organic cations plays a key role in constructing LDMHPs’ structures.

**Table 1:** Summary of reported organic cations of LDMHPs

Dimension^a)^	Formula	Connecting type	Fabrication	Peak [nm]	FWHM [nm]	PLQE [%]
1‐0D^[^ ^30^ ^]^	(Ph_4_P)_2_SbCl_5_	N/A	Antisolvent method	648	136	87
2‐0D^[^ ^29^ ^]^	(C_9_NH_20_)_2_SbCl_5_	N/A	Antisolvent method	590	119	98± 2
2‐0D^[^ ^31^ ^]^	(C_9_NH_20_)_7_(PbCl_4_)Pb_3_Cl_11_	Face‐sharing	Antisolvent method	470	84	83
2‐0D^[^ ^27^ ^]^	(C_9_NH_20_)_2_S*_n_* Br_4_	N/A	Solution method	695	146	46
2‐0D^[^ ^12^ ^]^	(bmpy)_9_[ZnCl_4_]_2_[Pb_3_Cl_11_]	Face‐sharing	Antisolvent method	470	≈50	83
3‐0D^[^ ^33^ ^]^	(C_4_H_8_N_2_H_4_)_2_PbBr_6_·2H_2_O	Corner‐sharing	Cooling crystallization	N/A	N/A	N/A
4‐0D^[^ ^34^ ^]^	(C_13_H_19_N_4_)_2_PbBr_4_	N/A	Antisolvent method	460	66	40
5‐0D^[^ ^35^ ^]^	(C_6_N_2_H_16_Cl)_2_SnCl_6_	N/A	Solution method	450	125	8.1
6‐0D^[^ ^12^ ^]^	(C_3_N_3_H_11_O)_2_PbBr_6_·4H_2_O	N/A	Antisolvent method	568	200	9.6
7‐1D^[^ ^36^ ^]^	(C_6_H_8_N_3_)_2_Pb_2_Br_10_	Corner‐sharing	Cooling crystallization	580	≈200	9
8‐1D^[^ ^37^ ^]^	[(AD)Pb_2_Cl_5_]	Corner‐sharing	Solution method	533	N/A	7.45
9‐1D^[^ ^38^ ^]^	[Nmethyldabconium]PbI_3_	Face‐sharing	Solution evaporation	469	N/A	N/A
10‐1D^[^ ^39^ ^]^	(C_6_H_13_N_4_)_3_Pb_2_Br_7_	Face‐sharing	Antisolvent method	580	158	7
11‐1D^[^ ^40^ ^]^	C_5_H_14_N_2_PbCl_4_·H_2_O	Edge‐sharing	Cooling crystallization	412, 617	N/A	N/A
12‐1D^[^ ^41^ ^]^	[C_6_H_14_N]PbI_3_	Face‐sharing	Solution evaporation	675	N/A	N/A

^a)^ The figures (1–12) highlighted in blue refers to the corresponding organic cations shown in Figure 4

### 2.2 Metal Ions

Most 2D perovskites are in (NH_4_RNH_4_)MX_4_ and (RNH_4_)_2_MX_4_ structures, where M is usually a divalent metal, e.g., Pb^2+^, Sn^2+^, Ge^2+^, Mn^2+^, Fe^2+^, Co^2+^, Cu^2+^. Here, Sn^2+^ and Ge^2+^ are often introduced to replace toxic Pb^2+^ in perovskites, but their stability is very poor, as the Sn^2+^ and Ge^2+^ ions are easily oxidized to Sn^4+^ and Ge^4+^.^[^ ^49^ ^]^ Recently, the Cu^2+^ based perovskites have attracted attention due to their good stability, such as the 2D 100‐oriented perovskite MA_2_CuCl_4−_ *_x_* Br*_x_* prepared by MA cations.^[^ ^54^ ^]^ In addition, a series of 2D perovskites with different organic cations including (C_4_H_9_NH_3_)_2_CuBr_4_,^[^ ^55^ ^]^ (C_5_H_9_NH_3_)_2_CuBr_4_,^[^ ^56^ ^]^ (C_6_H_5_CH_2_CH_2_NH_3_)_2_CuCl_4_,^[^ ^57^ ^]^ and (C_4_H_9_NH_3_)_2_MCl_4_,^[^ ^58^ ^]^ and the synthesis and characterization of (C_3_H_7_NH_3_)_2_MCl_4_ (M = Mn, Cu, Cd, Pb),^[^ ^59^ ^]^ (CnH_2_ *_n_*_+1_NH_3_)_2_MCl_4_ (M = Mn, Fe, Co, Cu, Zn, and *n* = 2, 4, 6, 8, 10, 12), ^[^ ^60^ ^]^ etc. have been reported. Recently, the layered double perovskite

**Figure 5:**
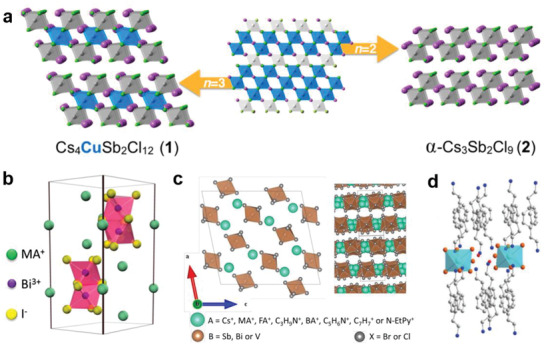
a) The regulation of layers is realized in 2D double perovskite Cs_4_CuSb_2_Cl_12_. Reproduced with permission.^[^ ^50^ ^]^ Copyright 2018, Wiley‐VCH. b) Structure of 0D perovskites (CH_3_NH_3_)_3_Bi_2_I_9_. Reproduced with permission.^[^ ^51^ ^]^ Copyright 2017, American Chemical Society. c) Structure of 0D perovskites ABiB_6_. Reproduced with permission.^[^ ^52^ ^]^ Copyright 2018, American Chemical Society. d) Lead‐free 0D double perovskite (C_8_NH_12_)_4_Bi_0.57_Sb_0.43_Br_7_·H_2_O. Reproduced with permission. ^[^ ^53^ ^]^ Copyright 2019, Wiley‐VCH.

Cs_4_CuSb_2_Cl_12_ with two metal ions (Cu^2+^ and Sb^3+^) was synthesized for the first time.^[^ ^50^ ^]^ As shown in **Figure** **5**a, the number of inorganic layers regulation of this material has been realized, which initiated a new chemical component design strategy to regulate the layers of 111‐oriented 2D perovskites. It's worth mentioning that Bi metals formed the 0D structures, such as (CH_3_NH_3_)_3_Bi_2_I_9_ (Figure 5b) with Bi^3+^,^[^ ^51^ ^]^ and ABiBr_6_ (A = Cs^+^, MA^+^, FA^+^, C_3_H_9_N^+^, BA^+^, C_5_H_6_N^+^, C_7_H_7_^+^, *N*‐EtPy^+^) with Bi^5+^ (Figure 5c).^[^ ^52^ ^]^ It has been reported that for 0D double perovskite (C_8_NH_12_)_4_Bi_0.57_Sb_0.43_Br_7_·H_2_O (Figure 5d),^[^ ^53^ ^]^ the individual [BiBr_6_]^3−^ and [SbBr_6_]^3−^ octahedrons are completely isolated and surrounded by large organic C_8_H_12_N^+^, and so they have exhibited a great stability.

**Figure 6:**
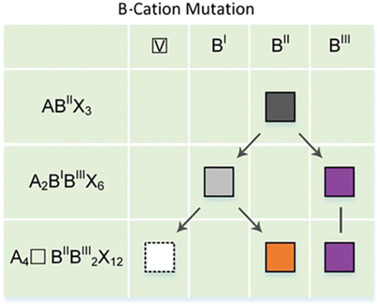
Design schematic diagram of cation mutations from simple perovskites to double perovskites and quadruple perovskites. Reproduced with permission.^[^ ^61^ ^]^ Copyright 2019, American Chemical Society.

The diversified options of metal ions can not only construct the double perovskites,^[^ ^9^ ^]^ but also theoretically design the structure of quadruple perovskites. For example, Lin et al. propose a strategy to design quadruple perovskites by introducing heterovalent cation to form double perovskites. And these two stable quadruple perovskite halides of Cs_4_CdSb_2_Cl_12_ and Cs_4_CdBi_2_Cl_12_ with vacancy‐ordered structures were successfully synthesized by solvent heat method and exhibited broadband emissions.^[^ ^61^ ^]^ **Figure** **6** shows the design diagram of quadruple perovskite, the quadruple perovskites provide a promising design method for structure regulation of perovskites.

### 2.3 Halogens

**Figure 7:**
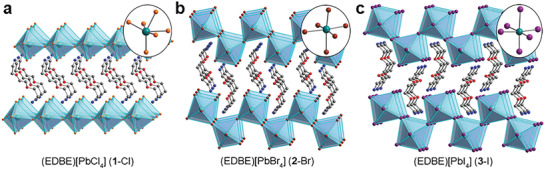
Structure of (EDBE)[PbX_4_] (X = Br and Cl) crystal structure. Reproduced with permission.^[^ ^17^ ^]^ Copyright 2014, American Chemical Society.

Halogens have great influence on the structures of LDMHPs, too. For example, Dohner et al. reported 2D perovskites (EDBE)[PbX_4_] (X = Cl^−^, Br^−^, I^−^)^[^ ^17^ ^]^ with white emissions. Crystal structures of these materials are shown in **Figure** **7**, in which the Pb‐Cl is a 100‐oriented structure. When the lead halide octahedron is deformed due to the different coordination environment of Pb^2+^, the 2D perovskites of Pb‐Br and Pb‐I became 110‐oriented structures. Kanatzidis et al. reported 2D hybrid perovskites of EA_4_Pb_3_X_10_ (X = Cl^−^, Br^−^),^[^ ^10^ ^]^ and when the halogen is I^−^, it did not form an analogue with EA_4_Pb_3_X_10_ (X = Cl^−^, Br^−^), but a 1D face‐sharing perovskite.

**Figure 8:**
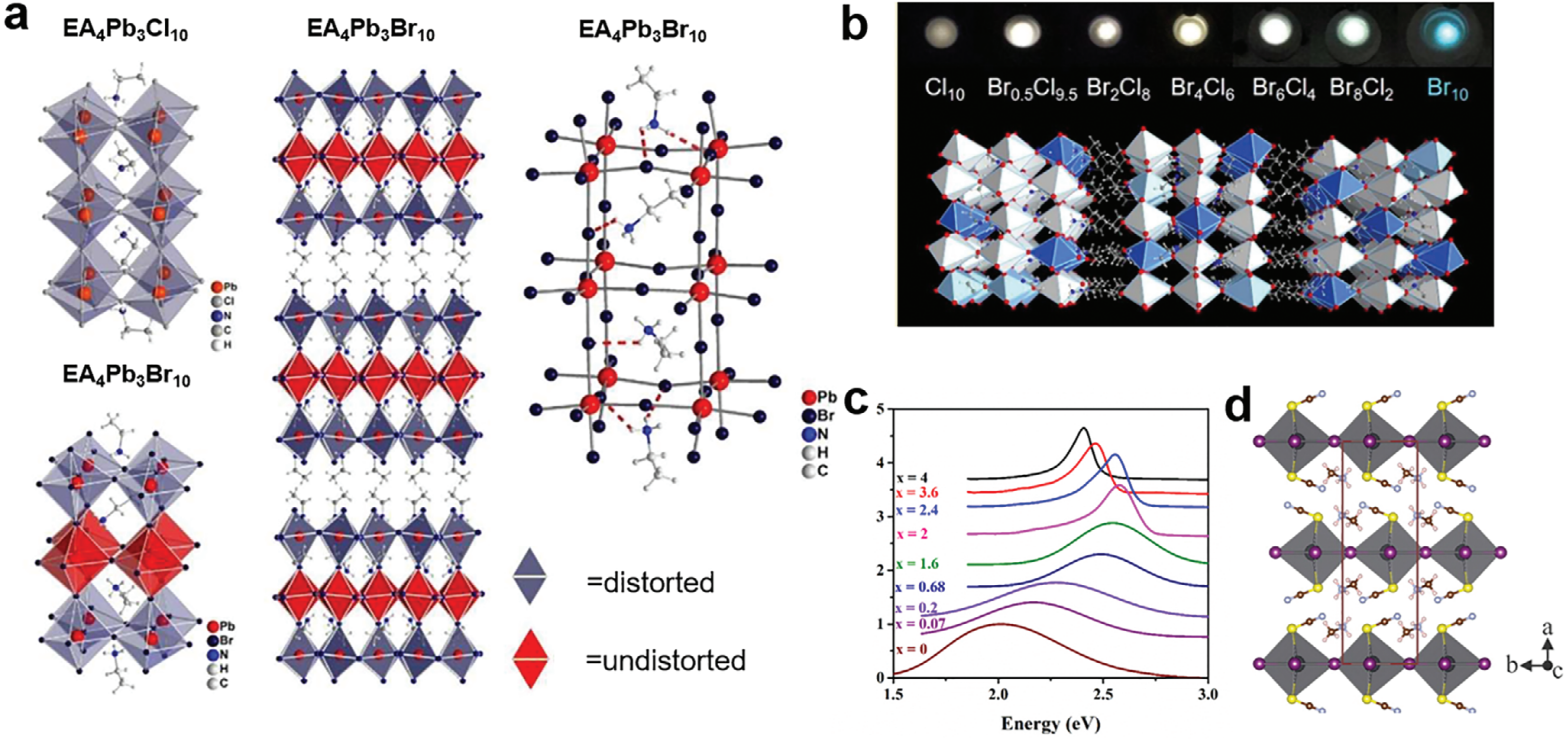
a) Structure diagram of EA_4_Pb_3_X_10_ (X = Br and Cl) (where the inner layers are “undistorted” octahedra and the outer layers are composed of “distorted” octahedra). b) The mixed halogen EA_4_Pb_3_Br_10−_ *_x_* Cl*_x_* (*x* = 0, 2, 4, 6, 8, 9.5, 10) crystals. a,b) Reproduced with permission.^[^ ^10^ ^]^ Copyright 2019, American Chemical Society. c) PL of (C_6_H_11_NH_3_)_2_[PbBr_4−_ *_x_* I*_x_*] vary with the content of I. Reproduced with permission.^[^ ^63^ ^]^ Copyright 2017, Elsevier B.V. d) Structure of 2D perovskite (CH_3_NH_3_)_2_Pb(SCN)_2_I_2_. Reproduced with permission.^[^ ^64^ ^]^ Copyright 2017, Royal Society of Chemistry.

Halogens affect structure distortions of LDMHPs significantly. As the ion radius of Cl^−^ (1.67 Å) is less than that of Br^−^ (1.82 Å), it does not accommodate the EA^+^ cation well and so the EA_4_Pb_3_Cl_10_ has highly distorted structure.^[^ ^62^ ^]^ For EA_4_Pb_3_Br_10_, the intermediate layer of Pb‐Br is of regular structure, while the outer layer Pb‐Br has obvious structural distortion (**Figure** **8**a). The distortion of intrinsic structure affects the luminescence characteristics of LDMHPs. That the effect of halogen replacement on structure distortion and luminescence properties have many examples and broadband emission is strongly associated with distorted degrees of lattices in LDMHPs. Mao et al.^[^ ^10^ ^]^ found that different distortion levels in EA_4_Pb_3_Cl_10_ (large distortion) versus EA_4_Pb_3_Br_10_ (small distortion) and EA_4_Pb_3_Cl_10_ has a broadband white‐light emission, while EA_4_Pb_3_Br_10_ has a narrow blue emission. By further tuning the ratio of chlorine/bromine for EA_4_Pb_3_Br_10−_ *_x_* Cl*_x_* (*x* = 0, 2, 4, 6, 8, 9.5, 10), they found that two of the intermediate compounds (*x* = 8 and 9.5) have more optimized white‐light emissions than that of pure EA_4_Pb_3_Cl_10_ (Figure 8b). Yangui et al.^[^ ^63^ ^]^ studied (C_6_H_11_NH_3_)_2_[PbBr_4_] and (C_6_H_11_NH_3_)_2_[PbI_4_] with similar structures, in which the [PbI_6_]^4−^ is more regular than [PbBr_6_]^4−^ octahedron. Figure 8c shows the PL variation of (C_6_H_11_NH_3_)_2_[PbBr_4−_ *_x_* I*_x_*] with mixed halogens. The full width at half‐maximum (FWHM) of PL gradually widened with the decrease of I^−^ content due to the difference of structure distortions. Furthermore, halogens can also tune structure distortion through hydrogen bonding formed between organic cations and halide anions. Here, different electronegative properties of halogens lead to different strength of hydrogen bonding force (where H‐Cl > H‐Br > H‐I), determining the orientation of organic cation^[^ ^18^ ^]^ in interlayer of metal halides and caused different distortion degrees of lead halide octahedron. Furthermore, the halogens can be replaced by other anions, such as the SCN^−^ which has a similar radius with I^−^. The 100‐oriented 2D perovskite (CH_3_NH_3_)_2_Pb(SCN)_2_I_2_^[^ ^64^ ^]^ can be obtained by partially replacing the I^−^ with SCN^−^. Its structure is shown in Figure 8d, in which the lead coordination octahedron is constructed by four equatorial I^−^ and two axial SCN^−^ coordination. Due to the polarity of SCN^−^, the octahedron is distorted by Pb─S bonds.^[^ ^65^ ^]^

### 2.4 Hydrogen Bonding

**Figure 9:**
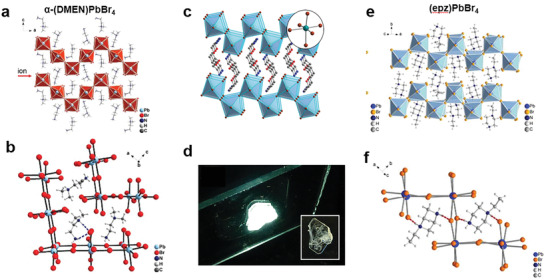
a) The 3 × 3 corrugated layer structure of *α*‐(DMEN)PbBr_4_ and b) its hydrogen bonds schematic diagram. a,b) Reproduced with permission.^[^ ^45^ ^]^ Copyright 2017, American Chemical Society. c) Structure of corrugated 2D perovskite (*N‐*MEDA)[PbBr_4_]. d) The perovskite (*N‐*MEDA)[PbBr_4_] under the irradiation of ultraviolet lamp shows bright white light. c,d) Reproduced with permission.^[^ ^17^ ^]^ Copyright 2014, American Chemical Society. e) Structure of (epz)PbBr_4_ and f) hydrogen bonding diagram. e,f) Reproduced with permission.^[^ ^66^ ^]^ Copyright 2018, American Chemical Society.

The all‐inorganic Cs‐M‐X structures (M = Pb^2+^, Sn^2+^, Ge^2+^ etc., X = Cl^−^, Br^−^, I^−^) are mainly constructed by electrostatic effects between negatively charged metal halide octahedrons and positively charged Cs^+^. For organic–inorganic hybrid perovskites, organic cations contain one or more terminal amines in which H atoms interacting with halogens on inorganic octahedrons by hydrogen bonding. For example, the 110‐oritented 2D perovskites have been widely studied recently due to their excellent white emission properties, and their unique corrugated structures are stabilized by hydrogen bonding. For example, Kanatzidis et al.^[^ ^45^ ^]^ reported the corrugated 2D perovskite (DMEN)PbBr_4_, as is shown in **Figure** **9**a. Its 3 × 3 corrugated layer configuration is stabilized by hydrogen bonding. With H atoms on the primary and secondary amines of organic cations forming hydrogen bonding with Br atoms, the inorganic layer folds ≈90°, producing the strongly corrugated structure (Figure 9b). In addition, many other corrugated 2D structures, such as (*N*‐MEDA)[PbBr_4_]^[^ ^17^ ^]^ which is shown in Figure 9c have been reported. It exhibits a white emission upon UV light with a high PLQE of 9% (Figure 9d). (Epz)PbBr_4_ (Figure 9e)^[^ ^66^ ^]^ is another example to demonstrate the role of hydrogen bonding in stabilizing the 110‐oriented 2D perovskites (Figure 9f).

**Figure 10:**
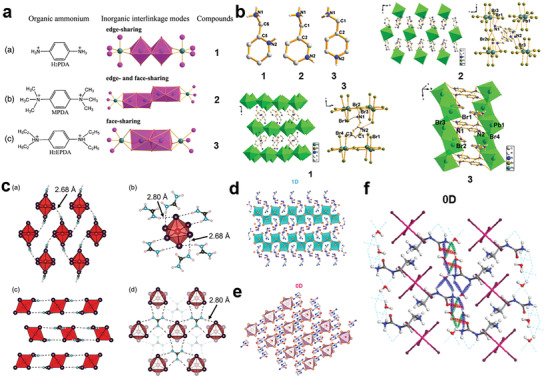
a) Different kinds of amines, primary amines, secondary amines, tertiary amines constructed 1D structures with different interlinkage modes. Reproduced with permission.^[^ ^43^ ^]^ Copyright 2011, Elsevier B.V. b) Organic cations at different sites and constructed 2D structure (H22‐AMP)PbBr_4_(1), 0D structure (H23‐AMP)_2_PbBr_6_(2), and 1D structure (H24‐AMP)PbBr_4_ (3). Reproduced with permission.^[^ ^67^ ^]^ Copyright 2007, Elsevier Masson SAS. c) Structure of (GUA)_2_PtI_6_, (FA)_2_PtI_6_ and their hydrogen bonds schematic diagram. Reproduced with permission.^[^ ^68^ ^]^ Copyright 2018, American Chemical Society. Structures of d) 1D (C_3_N_3_H_10_O)(C_3_N_3_H_11_O)_2_Pb_2_Br_9_ and e) 0D (C_3_N_3_H_11_O)_2_PbBr_6_·4H_2_O, respectively. f) View of hydrogen‐bonding in organic cations and water of the bulk 0D lead bromide crystal. Reproduced with permission.^[^ ^12^ ^]^ Copyright 2019, Springer Nature.

In fact, the formation of hydrogen bonding plays a decisive role in regulating the orientation and conformation of inorganic skeleton. Different amines, such as primary amines, secondary amines, tertiary amines all can construct metal halides 1D structures with different interlinkage modes (**Figure** **10**a),^[^ ^43^ ^]^ and the organic cations 1, 2, and 3 containing different N‐H sites which are adjacent, interphase and pair sites constructed 2D, 1D, and 0D configurations, respectively (Figure 10b).^[^ ^67^ ^]^ The structures of (GUA)_2_PtI_6_ and (FA)_2_PtI_6_^[^ ^68^ ^]^ are shown in Figure 10c, and here the discrete [PtI_6_]^4−^ octahedrons are connected by 3D hydrogen bonding network constructed by FA^2+^ and the 2D network constructed by GUA^2+^ cations, indicating that the hydrogen bonding force of organic cations affect the arrangement of inorganic [PtI_6_]^4−^ octahedrons. Recently, Cui et al. reported the structure transition from the corrugated 1D structure to 0D by adjusting hydrogen bonding (Figure 10d,e). By employing a unique urea‐amide cation containing H_4_N─C═O, which can form multiple hydrogen bonds between adjacent organic cations and inorganic skeleton. The corrugated 1D structure was stabilized by the hydrogen bonding of Pb─Br···H─N. When increase the concentration of amides in the growth process of crystals, the amides will form hydrogen bonds with H_2_O in precursor solution and adjacent cations to construct a large network (Figure 10f), which completely separated the [PbBr_6_]^4−^ octahedrons to form a 0D perovskite. The 1D and 0D perovskites both exhibited stable white emission, and the dimension reduction from 1D to 0D increased the PLQE as much as five times, providing a new strategy to regulate the structure and improve the luminescence performance by regulating hydrogen bonding forces in MHPs.^[^ ^12^ ^]^

### 2.5 Stoichiometric Ratio of Precursor Solution

**Figure 11:**
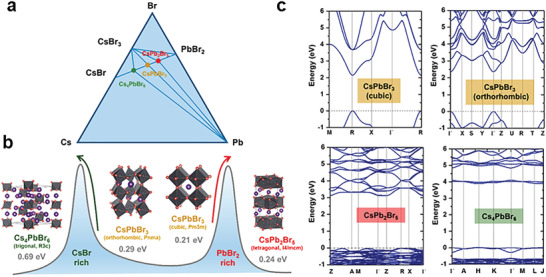
a) The ternary phase of Cs‐Pb‐Br, proportion of precursor solution (CsBr and PbBr_2_). b) Conversion between (CsPbBr_3_, CsPb_2_Br, and Cs_4_PbBr_6_). c) Electron band structure of CsPbBr_3_, CsPb_2_Br, and Cs_4_PbBr_6_. Reproduced with permission.^[^ ^11^ ^]^ Copyright 2018, American Chemical Society.

For all‐inorganic Cs‐Pb‐X perovskite crystals, different structure dimensions can be easily regulated by changing the stoichiometric ratio of precursor solution. For example, a ternary phase diagram of 1D Cs_4_PbBr_6_, 2D CsPb_2_Br_5_, and 3D CsPbBr_3_ is shown in **Figure** **11**a, in which dimension regulation is achieved by stoichiometric ratio (CsBr:PbBr_2_) control. As shown in Figure 11b, the traditional 3D structure of CsPbBr_3_ has two phases, cubic phase (Pm3m) and orthorhombic phase (Pnma). When PbBr_2_ is abundant, 2D structure CsPb_2_Br_5_ is formed,^[^ ^69^ ^]^ and Cs^+^ ions appear between the inorganic layers. In contrast, when CsBr is excess, 0D structure Cs_4_PbBr_6_ is formed, of which individual lead halide octahedrons are completely separated by Cs^+^.^[^ ^70^ ^]^ The bandgaps of these Cs‐Pb‐Br perovskites increase with the dimension decreasing (Figure 11c). Both CsPbBr_3_ and Cs_4_PbBr_6_ showed direct bandgap, while CsPb_2_Br_5_ showed indirect bandgap. The Cs‐Pb‐I perovskites can also construct different dimensions by controlling the stoichiometric ratio of CsI versus PbI_2_.

**Figure 12:**
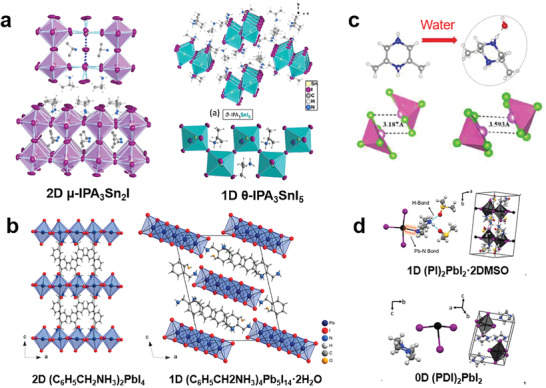
a) The structures of 2D *µ*‐IPA_3_Sn_2_I_7_ and 1D *θ*‐IPA_3_SnI. Reproduced with permission.^[^ ^71^ ^]^ Copyright 2017, American Chemical Society. b) The structures of 2D (C_6_H_5_CH_2_NH_3_)_2_PbI_4_ and 1D (C_6_H_5_CH_2_NH_3_)_4_Pb_5_I_14_·2H_2_O. Reproduced with permission.^[^ ^72^ ^]^ Copyright 2017, American Chemical Society. c) Diagram of organic cations ([C_6_N_2_H_16_]^+^ or [C_6_N_2_H_16_]·H_2_O^+^) and 0D [SbCl_5_] dimers. Reproduced with permission.^[^ ^73^ ^]^ Copyright 2020, Wiley‐VCH. d) The structures of 1D (PI)_2_PbI_2_·2DMSO and 0D (PDI)_2_PbI_2_. Reproduced with permission.^[^ ^74^ ^]^ Copyright 2020, American Chemical Society.

For organic–inorganic hybrid perovskites, a kind of organic cation usually constructed a kind of structure even the stoichiometric ratio of every elements in the precursor solution changed. However, when cations form different valence states in crystals, multiple dimensional structures, such as the 2D *µ*‐IPA_3_Sn_2_I_7_ and 1D *θ*‐IPA_3_SnI as shown in **Figure** **12**a can be built.^[^ ^71^ ^]^ On the other hand, different single crystal structures can also be obtained when H_2_O molecules grow into unit cells. For example, Cui et al. synthesized the 1D (C_3_N_3_H_10_O)(C_3_N_3_H_11_O)_2_Pb_2_Br_9_ with the ratio of C_3_N_3_H_9_O·2HBr : PbO = 3 : 2, and when the ratio increased to 2 : 1, H_2_O entered into the unit cell to form a 0D (C_3_N_3_H_11_O)_2_PbBr_6_·4H_2_O.^[^ ^12^ ^]^ Recently, Thomas T. M. et al. reported the well‐known 100‐oriented 2D (C_6_H_5_CH_2_NH_3_)_2_PbI_4_ phase with a corner‐sharing structure, and a new compound 1D edge‐sharing (C_6_H_5_CH_2_NH_3_)_4_Pb_5_I_14_·2H_2_O was generated when H_2_O incorporated into the crystal structure (Figure 12b).^[^ ^72^ ^]^ Furthermore, H_2_O molecules improved LDMHPs’ luminescent properties. Lin et al. has synthesized two new 0D (C_6_N_2_H_16_)SbCl_5_ and (C_6_N_2_H_16_)SbCl_5_·H_2_O, in which H_2_O molecules grew into lattices of (C_6_N_2_H_16_)SbCl_5_·H_2_O leading to the larger spatial distance between the [SbCl_5_] dimers (Figure 12c) and improving the PLQE of 57%.^[^ ^73^ ^]^ In addition, dimethylsulfoxide (DMSO) molecules also can be introduced to crystals to build different dimensional structures, such as the 0D (PDI)_2_PbI_2_ and 1D (PDI)_2_PbI_2_·2DMSO (Figure 12d).^[^ ^74^ ^]^

### 2.6 Temperature Effect

**Figure 13:**
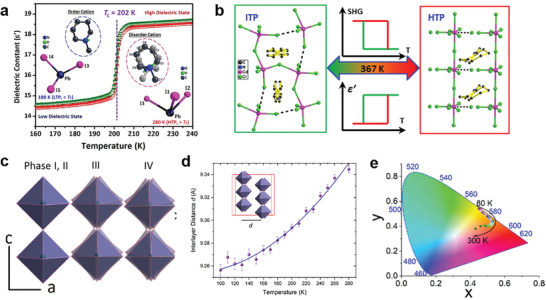
a) Phase transition and dielectric response of 1D MPIP perovskite structural phase transition induced by temperature.  Reproduced with permission.^[^ ^77^ ^]^ Copyright 2018, Royal Society of Chemistry. b) Structural phase transition of [C_6_H_11_NH_3_]_2_CdCl_4_ at 367 K. Reproduced with permission. ^[^ ^78^ ^]^ Copyright 2014, American Chemical Society. c) MHy_2_PbI_4_ underwent three structural phase transitions and the tilt degree of octahedral PbI_6_^4−^ at each phase. ^[^ ^14^ ^]^ d) The interval between lead iodide sheets varies with temperature.^[^ ^14^ ^]^ Copyright 2019, American Chemical Society. e) CIE coordinates of MHy_2_PbI_4_ at different temperatures. Reproduced with permission.^[^ ^14^ ^]^ Copyright 2019, American Chemical Society.

Perovskite materials usually undergo phase transition when the temperature changes, which are mainly caused by changes in configurations of organic cations. For example, the organic cations were disorderly arranged as the temperature increased (> 300 K) and the disordered point was called the melting temperature.^[^ ^75^ ^]^ The disordered cations led to sudden change of 2D interlayer distance and lattice parameters along the direction of organic cations.^[^ ^76^ ^]^ Recently, Zeb et al.^[^ ^77^ ^]^ synthesized a 1D perovskite crystal 1‐methylpiperidinium triiodoplumbate(II) (MPIP). They found the MPIP had an obvious phase transformation near the Tc = 202 K due to the ordered‐disordered transition of 1‐methylpiperidinium cation (**Figure** **13**a). In addition, Liao's group^[^ ^78^ ^]^ synthesized a 1D [C_6_H_11_NH_3_]_2_CdCl_4_ with high temperature dielectric response, due to it undergoes the phase transition at 367 K caused by the change of relative position of Cd atoms (Figure 13b).

**Figure 14:**
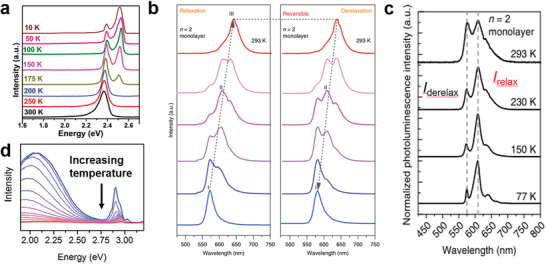
a) Changes of PL spectrum of stripped monolayer (C_4_H_9_NH_3_)_2_PbI_4_ with temperature. Reproduced with permission.^[^ ^80^ ^]^ Copyright 2018, American Chemical Society. b) 2D perovskite (*n* = 2) under laser irradiation, PL redshift, and then blueshift reversible process after laser annealing. Reproduced with permission.^[^ ^81^ ^]^ Copyright 2018, Springer Nature. c) Temperature‐dependent changes in PL intensity, highlighting changes in the relative intensity of relaxed peak. Reproduced with permission.^[^ ^81^ ^]^ Copyright 2018, Springer Nature. d) The broadband emission appears at low temperature in 100‐oriented 2D perovskite. Reproduced with permission.^[^ ^82^ ^]^ Copyright 2017, Royal Society of Chemistry.

Temperature not only affects LDMHPs’ structures, but also causes difference in their photoelectric properties. For example, Eric et al. studied the exciton characteristics of 2D (C_4_H_9_NH_3_)_2_PbI_4_ in a temperature range of 10–300 K.^[^ ^79^ ^]^ Under the drive of C_4_H_9_NH_3_^+^ rearrangement, phase transition occurred at around 250 K, corresponding to the decrease of lattice spacing and the increase of exciton binding energy. In addition, Sieradzki et al.^[^ ^14^ ^]^ synthesized a 2D (MHy_2_PbI_4_) as shown in Figure 13c. With the temperature decreasing, MHy_2_PbI_4_ underwent three structure phase transitions at 320, 298, and 262 K, respectively. The second phase transition at 298 K was related to the arrangement of MHy^+^, and this phase transition caused a significant change in the dielectric constant. As shown in Figure 13d, the interlayer spacing between the inorganic layers increased with the temperature increasing. In addition, the emission of MHy_2_PbI_4_ exhibited blueshift during cooling temperature from 300 to 80 K. It exhibited light yellow emission at 300 K and turned to yellow‐green emission at 80 K (Figure 13e). The few‐layer exfoliated 2D (C_4_H_9_NH_3_)_2_PbI_4_ underwent phase transition at ≈175 K, producing a new blueshift PL peak (**Figure** **14**a).^[^ ^80^ ^]^ It is worth mentioning that temperature has a great influence on the emission from relaxed states of LDMHPs. These materials usually have emission from relaxed states caused by lattice distortion or self‐trapped excitons (STEs). The PL intensity of relaxed states is more efficient than the free exciton (FE) at low temperatures.

Recently, RPP perovskite (CH_3_(CH_2_)_3_NH_3_)_2_(CH_3_NH_3_)*_n_*_−1_Pb_n_I_3_ *_n_*_+1_(*n*  = ≈1–4) has been reported,^[^ ^81^ ^]^ and it undergoes structure distortion under laser irradiation, resulting in a relaxed redshifted emission (Figure 14b). The relaxation peak increased sharply with the temperature decreasing (Figure 14c). In addition, the STE emission intensity also increased as the temperature decreased. Some 2D 100‐oriented perovskites can observe STE emissions at low temperature (Figure 14d).^[^ ^48^, ^82^ ^]^ When temperatures are high, the STEs are easy to detrap from STE states to FE states, and at low temperatures, the detrapping process is suppressed,^[^ ^82^ ^]^ Therefore, STEs emission gradually appears and increases at lower temperature.

### 2.7 Pressure Effect

**Figure 15:**
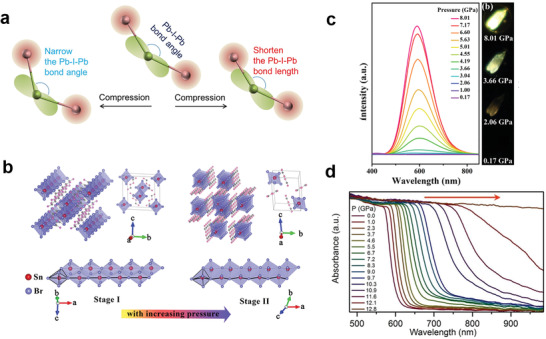
a) Schematic diagram of Pb─I─Pb bond under compression in (BA)_2_PbI_4_. Reproduced with permission.^[^ ^86^ ^]^ Copyright 2018, AmericanChemical Society. b) 1D structure distortion caused by pressure. c) The relationship between PL spectra of C_4_N_2_H_14_SnBr_4_ and pressure, and luminescence photographs at different pressure points. b,c) Reproduced with permission.^[^ ^13^ ^]^ Copyright 2019, American Chemical Society. d) Optical absorption spectrum of Cs_3_Bi_2_I_9_ at high pressure. Reproduced with permission.^[^ ^87^ ^]^ Copyright 2018, Wiley‐VCH.

Exerting high pressure to the material is an effective way to regulate structures and photoelectric properties of LDMHPs. For example, Liu et al.^[^ ^83^ ^]^ reported that 2D (BA)_2_(MA)*_n_*_−1_Pb*_n_* I_3_ *_n_*_+1_ (*n* = 3) reduced the bandgap by 8.2% after increasing the pressure. This is because external pressure enhanced coupling between s orbitals of Pb atoms and p orbitals of I atoms. Yin et al.^[^ ^84^ ^]^ studied the mechanically exfoliated 2D layers of (C_4_H_9_NH_3_)_2_PbI_4_ under pressure. As shown in **Figure** **15**a, under initial compression from 1 atm to 0.5 GPa, the slight blueshifts of bandgap happened due to the decreased Pb─I─Pb bond angles. Perovskite lattices were compressed when pressure continued to increase, and the bond length of Pb─I─Pb inevitably decreased. This increased the overlap of electronic wave function of Pb and I atoms, and finally caused bandgap redshifts.

Shi's team^[^ ^13^ ^]^ synthesized a unique 1D organic tin bromide perovskite C_4_N_2_H_14_SnBr_4_. The 1D inorganic chain deformed under the transition from monoclinal phase to triclinic phase (Figure 15b), and the exciton binding energy increased, resulting in the enhancement of PL

intensity (Figure 15c). This work revealed a potential application of supercharging to improve the luminescence performance of LDMHPs. Zhang et al.^[^ ^85^ ^]^ studied the PL properties of 0D Cs_3_Bi_2_I_9_ under high pressure. As shown in Figure 15d, under high pressure, the bandgap of Cs_3_Bi_2_I_9_ continued to become narrow, and finally reached the optimal value. In addition, at atmospheric pressure, the isolated [Bi_2_I_9_]^3−^ octahedron exhibited only weak emission upon excitation. But it exhibited significant enhancement of PL intensity about ten times under relatively high pressure (< 1 GPa) due to the increased exciton binding energy.

**Figure 16:**
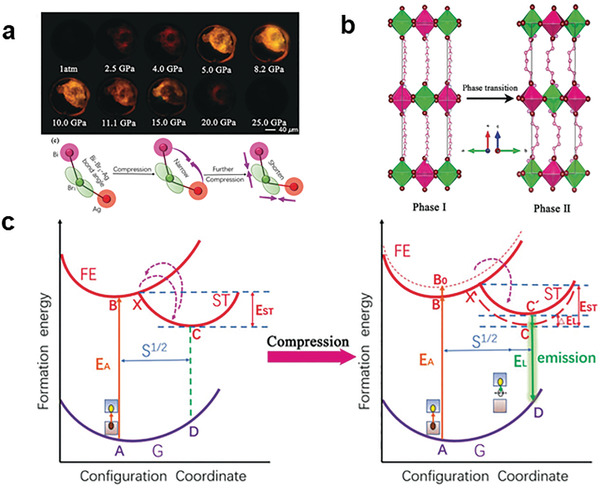
a) The photomicrograph shows that the strength of PL varies with the increase of pressure. b) The compressive structural transformation of (BA)_4_AgBiBr_8_ crystals (the pink and green octahedrons represent [AgBr_6_] and [BiBr_6_] octahedrons, respectively). c) Schematic diagram of the pressure‐induced luminescence mechanism of self‐trapped exciton. Reprinted with permission. ^[^ ^86^ ^]^ Copyright 2019, Wiley‐VCH.

It is worth mentioning that external pressure could distort the structure of the LDMHP to produce its STE emission. Recently, it has been reported that the pressure effect on regulating the structure distortion achieving the STE emission of (BA)_4_AgBiBr_8_.^[^ ^86^ ^]^ With the increasing pressure, the emission of (BA)_4_AgBiBr_8_ changed from a nonluminescent state under atmospheric pressure to a state with obvious fluorescence at 2.5–25.0 GPa (**Figure** **16**a). The sample completely transformed to a new structure at 2.1 GPa (Figure 16b). In addition, the new structure gradually distorted and its crystallinity reduced upon compression. Finally, a completely lower crystallinity phase formed at around 25.0 GPa, which led to the quenching of the luminescence. The luminescence mechanism is shown in Figure 16c. Under atmospheric conditions, the STEs were easy to detrap and return to FE states. When the pressure increases, the structure distortion of (BA)_4_AgBiBr_8_ deepens its self‐trapped states, which enhanced the energy barrier to avoid the detrap of excitons. And the bright emission from self‐trapping realized.

## 3 Luminescence Applications

### 3.1 Light‐Emitting Diodes (LEDs)

Due to the size effect and significant differences in dielectric constants between the organic and inorganic parts, excitons in LDMHPs are spatially confined. This suppresses the separation of excitons and greatly improves the radiation recombination efficiency. Therefore, organic–inorganic LDMHPs are considered to be good candidate materials for stable LEDs with high‐efficiency. Most 2D perovskite electroluminescent layers can be fabricated via the one or two‐step fast crystallization spin‐coating method using precursors. **Table** **2** shows a performance summary of LEDs based on recent 2D MHPs. However, 1D and 0D fail to be fabricated by solution method, which is the main reason for no 1D and 0D electroluminescent diodes. Therefore, unlike “structure‐level” 1D nanowire/nanorod perovskites and 0D nanocrystal perovskites, 1D and 0D organic–inorganic MHPs at the molecular level have not been used in the electroluminescent layer.^[^ ^87^ ^]^

**Table 2:** Performance summary of reported LDMHPs‐based LEDs

Formula	Device fabrication	EQE%	Stability
PEA_2_MA*_n_*_−1_Pb*_n_* Br_3_ *_n_*_+1_(*n* = 5)^a)^ ^[^ ^99^ ^]^	Spin‐coating using precursors	7.4	N/A
BA_2_MA*_n_*_−1_PbnI_3_ *_n_*_+1_(*n* = 11)^b)^ ^[^ ^100^ ^]^	Spin‐coating using precursors	10.4	No degradation after storage for 8 months in N_2_
(NMA)_2_CsPb_2_I_7_ ^c)^ ^[^ ^101^ ^]^	Spin‐coating using precursors	3.7	50% after 5 h under operation
(EA)_2_(MA)*_n_*_−1_Pb*_n_* Br_3_ *_n_*_+1_ ^d)^ ^[^ ^102^ ^]^	Spin‐coating using precursors	2.6	N/A
PA_2_(CsPbBr3)*_n_*_−1_PbBr_4_ ^e)^ ^[^ ^103^ ^]^	Spin‐coating using precursors	3.6	50% after around 30 min under operation
(NMA)_2_Cs*_n_*_−1_Pb*_n_* I_3_ *_n_*_+1_^[^ ^104^ ^]^	Spin‐coating using precursors	7.3	The EL emission peak does not change at different bias voltages
BA_2_FA_2_Pb_3_Br_10_^[^ ^43^ ^]^	Spin‐coating using precursors	14.64	The EL spectra retained up to 90% and 50% after 95 and 102 min
BA_2_(CsPbBr_3_)*_n_*_−1_PbBr_4_‐PEO^[^ ^105^ ^]^	Spin‐coating using precursors	8.42	50% after around 45 min under operation
(NMA)_2_FAPb_2_I_7_^[^ ^106^ ^]^	Spin‐coating using precursors	12.7	N/A
(TFA)_2_MA*_n_*_−1_Pb*_n_* Br_3_ *_n_* ^f)^ ^[^ ^107^ ^]^	Spin‐coating using precursors	N/A	No change after 2688 h in air
PEA_2_(FAPbBr_3_)*_n_*_−1_PbBr_4_(*n* = 3) ^[^ ^99^ ^]^	Spin‐coating using precursors	14.36	N/A
(BA)_2_(Cs)*_n_*_−1_[Pb*_n_* I_3_ *_n_*_+1_] ^[^ ^93^ ^]^	Spin‐coating using precursors	6.23	El spectral stability under operation
BA_2_DMA_1.6_Cs_2_Pb_3_Br_11.6_^[^ ^108^ ^]^	Spin‐coating using precursors	2.4	High spectrum stability at high voltage
PEA_2_DMA_1.2_Cs_2_Pb_3_Br_11.2_^[^ ^108^ ^]^	Spin‐coating using precursors	1.58	High spectrum stability at high voltage
PEA_2_(Rb_0.6_Cs_0.4_)_2_Pb_3_Br_10_^[^ ^109^ ^]^	Spin‐coating using precursors	1.35	No spectral change after 4 h annealing
CsPbCl_0.9_Br_2.1_ with PEABr^[^ ^110^ ^]^	Spin‐coating using precursors	5.7	El spectral stability under operation
(PEA)_2_(CH_3_NH_3_)*_m_*_−1_Pb*_m_* Br_3_ *_m_*_+1_ (*m* = 3) ^[^ ^96^ ^]^	Spin‐coating using precursors	4.98	Superior stability under exposure to light and moisture
(BIZ)_2_Mn_0.23_Pb_0.77_I_4_ ^g)^ ^[^ ^111^ ^]^	Spin‐coating using precursors	0.045	Good color stability
PEA_2_Cs_2_Pb_3_Br_10_ (with QDs) ^[^ ^112^ ^]^	In situ fabrication of QDs films	8.1	Over 1 h at a current density of 10 mA cm^−2^ of continuous operation
PEA_2_MA_2_Pb_3_Br_10_^[^ ^113^ ^]^	Spin‐coating using precursors	9.2%±1.43	N/A
(BA)_2_CsPb_2_Br_7_ (single crystals) ^[^ ^114^ ^]^	Spin‐coating using precursors	0.7	N/A
(BA)_2_Cs_2_Pb_3_Br_10_ (single crystals) ^[^ ^114^ ^]^	Spin‐coating using precursors	1.1	N/A

^a)^ PEA, C_6_H_5_C_2_H_4_NH_3_^+^

^b)^ BA, CH_3_(CH_2_)_3_NH_3_^+^

^c)^ NMA, 1‐naphthylmethyl ammonium

^d)^ EA, C_2_H_5_NH_3_^+^

^e)^ PA, C_3_H_7_NH_3_^+^

^f)^ TFA, C_6_H_2_F_3_NH_3_^+^

^g)^ BIZ, benzimidazolium.

**Figure 17:**
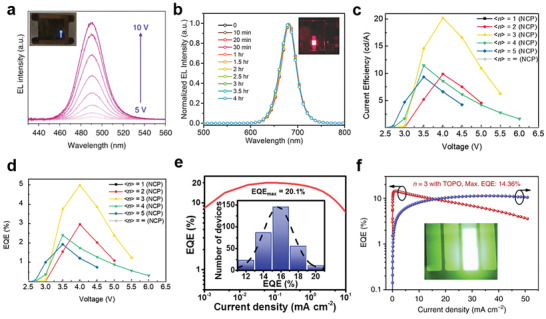
a) EL spectra of LEDs operating under different voltage. Inset is a photograph of the device. Reproduced with permission. ^[^ ^92^ ^]^ Copyright 2018, Springer Nature. b) EL spectral stability under 3.5 V continuous voltage operation. Inset is a photograph of the device. Reproduced with permission.^[^ ^93^ ^]^ Copyright 2018, Wiley‐VCH. c) Current efficiency–voltage curve of the LED device. d) External quantum efficiency–voltage curve of the LED device. c,d) Reproduced with permission.^[^ ^96^ ^]^ Copyright 2019, Wiley‐VCH. e) EQE‐current density curve of the LEDs (peak EQE = 20.1%). Inset is the peak EQE histogram of 320 devices. Reproduced with permission.^[^ ^2^ ^]^ Copyright 2018, Springer Nature. f) EQE of the champion device of PEA_2_(FAPbBr_3_)*_n_*_−1_PbBr_4_ (*n* = 3 composition) with TOPO as passivation layer. Inset is the EL image of the green LED. Reproduced with permission.^[^ ^99^ ^]^ Copyright 2018, Springer Nature.

In order to prepare color‐adjustable LEDs, two main strategies have been tried so far. One is to rely on mixed halides,^[^ ^88^ ^]^ and the other is to control quantum well structures.^[^ ^89^ ^]^ However, ion migration and phase separation are prone to occur under light and electric fields in LED devices with mixed halide perovskites.^[^ ^90^ ^]^ resulting in the changes in electroluminescent color during device operation.^[^ ^91^ ^]^ Controlling the quantum well structure to obtain quasi‐2D perovskite is another effective method to obtain the target color. The LEDs produced by this method have a very stable electroluminescence spectrum. For example, Xing et al.^[^ ^92^ ^]^ introduced a short ligand (*iso*‐*pro*‐pylammonium, IPA) to partially replace the long ligand (PEA) to synthesize a quasi‐2D perovskites. The growth of *n* = 2, 3, 4 phases become dominant, thereby adjusting the crystallization of the quasi‐2D perovskites. The fabricated LEDs devices reached a maximum brightness of 2480 cd m^−2^ at 490 nm and showed stable sky blue emission under high operating voltage (**Figure** **17**a). In addition, Tian et al.^[^ ^93^ ^]^ prepared a quasi‐2D perovskites /poly(ethylene oxide) (PEO) composite films by blending metal halide perovskites with polymer, and used it as a light‐emitting layer to prepare a good spectral stability bright red perovskite LEDs (Figure 17b).

However, the insulating properties of the large cations limit the carrier transport in 2D perovskites, thereby reducing the radiation recombination rate of quasi‐2D perovskite based LEDs.^[^ ^94^ ^]^ It has been reported that inorganic alkali metals with small ionic radius such as Na^+^ was used to replace large organic cations partly in perovskite layers to improve the carrier transport.^[^ ^95^ ^]^ Another way is to ensure the good connection of the inorganic layer through the optimized nanocrystalline pinning (NCP) process, which can effectively promote the carrier transfer in the LED devices of quasi‐2D perovskites. For example, Lee et al.^[^ ^96^ ^]^ used the optimized NCP process to modulate the Ruddelsden–Popper (RP) phase of (PEA)_2_(CH_3_NH_3_)*_n_*_−1_Pb*_n_* Br_3_ *_n_*_+ 1_ nanostructure, and their RP phase units oriented randomly. This modulated nanostructure can increase the contact between adjacent quasi‐2D perovskite layers, and so carriers can be effectively transported through the connection interface between the inorganic layers, greatly improving the efficiency of RP quasi‐2D perovskite LEDs. When *n* = 3, they developed RP quasi‐2D perovskite LED devices with a maximum current efficiency of 20.18 cd A^−1^ (Figure 17c) and a maximum EQE of 4.98% (Figure 17d).

In addition, the conversion of 3D perovskites to quasi‐2D perovskites will introduce more defects on the surface or grain boundaries due to the reduction of crystal size, resulting in nonradiative recombination rate, which will also reduce the electroluminescence efficiency 2D perovskite LEDs.^[^ ^97^ ^]^ The intrinsic defects in the perovskite films can be passivated by introducing additives, or the interface modification methods can effectively reduce the defects in the perovskite films, thereby improving the radiation recombination rate in 2D perovskite LEDs.^[^ ^98^ ^]^ For example, Zhao et al.^[^ ^2^ ^]^ reported quasi‐2D and 3D (2D/3D) perovskite‐polymer (poly (2‐hydroxyethyl methacrylate)) heterostructure LEDs. The introduction of the polymer effectively eliminated the nonradiative recombination pathway, and the external quantum efficiency of the LED devices was as high as 20.1% (current density is 0.1–1 mA cm^−2^, Figure 17e). And Yang et al.^[^ ^99^ ^]^ investigated LEDs with high efficiency based on quasi‐2D PEA_2_(FAPbBr_3_)*_n_*_−1_PbBr_4_ (*n* = 3) as the emitting layer. External quantum efficiency (EQE) of the champion device with trioctylphosphine oxide (TOPO) passivation layer is up to 14.36% (Figure 17f).

### 3.2 Phosphors in Solid Lighting

**Figure 18:**
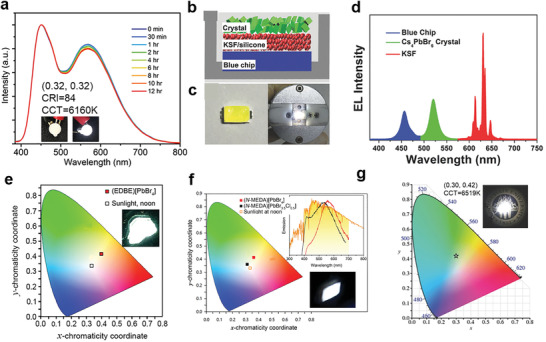
a) Emission stability and luminous performance of 0D (C_4_N_2_H_14_Br)_4_SnBr*_x_* I_6−_ *_x_* LED. Reproduced with permission.^[^ ^115^ ^]^ Copyright 2017, American Chemical Society. b) Schematic diagram of the configuration of the prototype device. c) A picture of surface mounted device (left) and the WLED operated at a forward bias current (right). d) EL spectrum of the prototype WLED device. b‐d) Reproduced with permission.^[^ ^116^ ^]^ Copyright 2018, Wiley‐VCH. e) CIE coordinates of (EDBE)(PbBr_4_) and sunlight at noon. Inset is a photograph of (EDBE)(PbBr_4_) under 365 nm irradiation. Reproduced with permission.^[^ ^17^ ^]^ Copyright 2014, American Chemical Society. f) CIE coordinates and emission spectrum of white‐light emitters and sunlight a noon. Inset is a photograph of (*N‐*MEDA)[PbBr_4_] under 380 nm irradiation. Reproduced with permission.^[^ ^46^ ^]^ Copyright 2014, American Chemical Society. g) CIE coordinate of (EDBE)PbBr_4_. Inset is an image of a UV‐pumped WLED. Reproduced with permission.^[^ ^124^ ^]^ Copyright 2016, Wiley‐VCH.

LDMHPs have larger exciton binding energies, which greatly increases the exciton recombination rate, resulting in LDMHPs with high photoluminescence quantum yield (PLQE). The Ma's group^[^ ^115^ ^]^ has reported an efficient broadband yellow‐emitting phosphors composed of 0D tin mixed halide perovskites (C_4_N_2_H_14_Br) _4_SnBr*_x_* I_6−_ *_x_* (*x* = 3). The phosphors had a full width half maximum of 126 nm (FWHM) and a photoluminescence quantum efficiency (PLQE) of about 85% due to the structural reorganization of the excited state. By mixing the yellow phosphors and commercial Eu‐doped barium magnesium aluminate blue phosphors (BaMgAl_10_O_17_: Eu^2 +^) with the weight ratio 1:4, a near‐perfect white emission with CIE coordinates of (0.32, 0.32), the color rendering index (CRI) of 84 and the correlated color temperature (CCT) value of 6160 K can be fabricated (**Figure** **18**a). UV‐pumped white LEDs was as high as 85. Zhong's group^[^ ^116^ ^]^ used the HBr‐assisted slow cooling method (SCM) to grow centimeter‐sized Cs_4_PbBr_6_ crystals with embedded CsPbBr_3_ nanocrystals, showing excellent green PL emission. Great green light‐emitting performance up to 97% of PLQE and excellent thermal stability made the materials an attractive candidate for white light‐emitting diodes (WLEDs) manufacturing. They also fabricated WLED devices by using green‐emitting crystals Cs_4_PbBr_6_ with CsPbBr_3_ nanocrystals, red‐emitting phosphor K_2_SiF_6_:Mn^4+^ (KSF), and blue‐emitting GaN chips. The optimized devices had a luminous efficiency of 151 lm W^−1^ at 20 mA and a chromaticity coordinate value of (0.331, 0.331) (Figure 18b–d).

Actually, white‐light‐emitting from a single emitter layer is very important for solid‐state lighting applications because it simplifies the device structure and avoids problems, such as self‐absorption and color instability in hybrid emitters and multiple emitters.^[^ ^46^ ^]^ Recently, a series of LDMHPs with 2D, 1D, and 0D structures have been reported. There is a variety of low‐dimensional single crystal growth strategies including but not limited to cooling crystallization method, antisolvent method and solution method. For 2D and 1D, adjacent octahedrons can be connected by corner‐sharing, edge‐sharing, and face‐sharing. They all exhibit broadband white‐light‐emitting and are applied to a single white‐emitting WLED phosphor. For example, The Ma's group^[^ ^19^ ^]^ also reported an 1D organic halide perovskites C_4_N_2_H_14_PbBr_4_ with the edge‐sharing octahedral lead bromide chains [PbBr_4_^2−^] that emitted bluish white‐light with a PLQE of 20%. To utilize the phosphor‐converted WLEDs for artificial lighting requirement, the CRI points of 95,^[^ ^117^ ^]^ the CCT of 5564 K,^[^ ^118^ ^]^ and the PLQE of 90%^[^ ^119^ ^]^ should be the standards. **Table** **3** gives a summary of LDMHPs phosphors used in WLED devices. Karunadasa and his colleagues^[^ ^17^, ^46^ ^]^ published 2D hybrid perovskites with 110‐oriented broadband white‐light‐emitting, namely (EDBE)PbBr_4_ (Figure 18e) and (*N‐*MEDA)PbBr_4_ (Figure 18f), which had broadband white‐light‐emitting due to the formation of self‐trapped excitons in the deformable lattice. Recently, Ma's group^[^ ^120^ ^]^ reported a high‐luminance micrometer‐scale corrugated 2D lead bromide perovskite (EDBE)PbBr_4_, its emission almost cover the entire visible spectrum, and its PLQE up to 18%. This perovskite phosphor can produce white‐light‐emitting under the irradiation of ultraviolet lamp. The CCT pumped by the 365 nm UV‐LED chip is 6519 K, and the CIE coordinates are (0.30, 0.42) (Figure 18g).

**Table 3:** Summary of reported LDMHPs phosphors used in white LED devices

Dimension^a)^	Formula	Connecting type	Fabrication	Peak [nm]	FWHM [nm]	PLQE [%]
21‐110‐2D^[^ ^45^ ^]^	*α*‐(DMEN)PbBr_4_	Corner‐sharing	Cooling crystallization	≈520	≈180	N/A
22‐110‐2D^[^ ^46^ ^]^	(*N‐*MEDA)[PbBr_4−_ *_x_* Cl*_x_*]	Corner‐sharing	Solution method	≈530	220	<1.5
23‐110‐2D^[^ ^47^ ^]^	C_6_H_13_C_l4_N_3_Pb	Corner‐sharing	Solution evaporation	573	220	<1
25‐110‐2D^[^ ^25^ ^]^	(PDA)_7_Pb_6_Br_26_	Corner‐sharing	Antisolvent method	671	265	6.7
27‐110‐2D^[^ ^17^ ^]^	(EDBE)[PbBr_4_]	Corner‐sharing	Solution method	573	215	9
27‐100‐2D^[^ ^17^ ^]^	(EDBE)[PbCl_4_]	Corner‐sharing	Solution method	538	208	2
20‐100‐2D^[^ ^10^ ^]^	(CH_3_CH_2_NH_3_)_4_Pb_3_ Br_10−_ *_x_* Cl*_x_*	Corner‐sharing	Solution method	359–450	150–228	N/A
24‐100‐2D^[^ ^48^ ^]^	(2meptH_2_)PbBr_4_	Corner‐sharing	Solution method	417	≈50	3.37
18‐1D^[^ ^12^ ^]^	(C_3_N_3_H_10_O)(C_3_N_3_H_11_O)_2_Pb_2_Br_9_	Corner‐sharing	Antisolvent method	530	218	1.7
25‐1D^[^ ^25^ ^]^	(EDA)_2_PbBr_6_	Corner‐sharing	Antisolvent method	523	138	9.1
17‐0D^[^ ^121^ ^]^	(C_4_N_2_H_14_I)_4_SnI_6_	N/A	Antisolvent method	620	188	75±4
17‐0D^[^ ^121^ ^]^	(C_4_N_2_H_14_Br)_4_SnBr_6_	N/A	Antisolvent method	570	105	95±5
6‐0D^[^ ^12^ ^]^	(C_3_N_3_H_11_O)_2_PbBr_6_·4H_2_O	N/A	Antisolvent method	568	218	9.6

^a)^ The figures highlighted in blue refers to the corresponding organic cations shown in Figure 4.

### 3.3 Scintillator and Transducer

**Figure 19:**
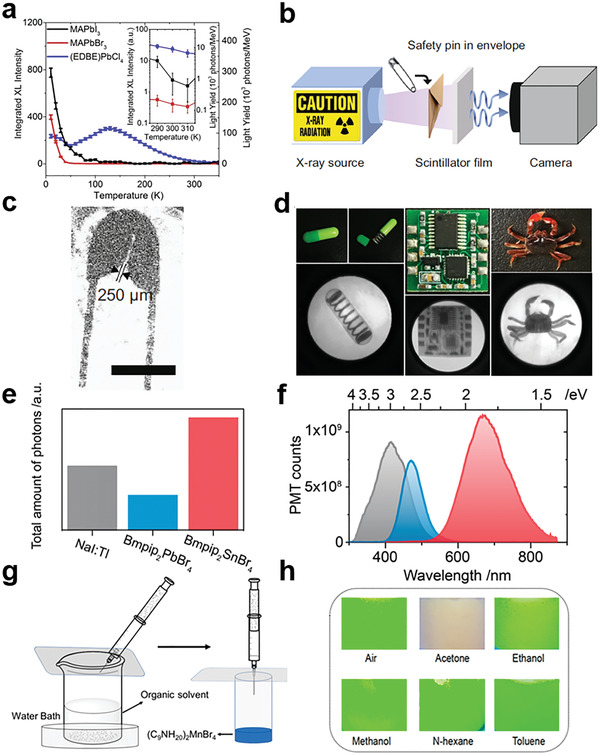
a) Temperature dependence of the light yields. The inset shows details of the curves from 290 to 310 K. Reproduced with permission.^[^ ^123^ ^]^ Copyright 2016, Spring Nature. b) Schematic diagram of X‐ray imaging setup using 1:1 Li‐(PEA)_2_PbBr_4_ as scintillator. c) X‐ray image of the safety pin using Li‐(PEA)_2_PbBr_4_ as scintillator. b,c) Reproduced with permission.^[^ ^126^ ^]^ Copyright 2020, Spring Nature. d) Photographs of a capsule containing a spring inside, a circuit board, a crab and the corresponding X‐ray images by X‐ray imaging. Reproduced with permission.^[^ ^127^ ^]^ Copyright 2020, American Chemical Society. e) The normalized total amount of photons comparison with the same X‐ray excitation. f) X‐ray fluorescence spectra under 50 kV Ag tube irradiation. Gray: NaI:TI commercial scintillator. Blue: Bmpip_2_PbBr_4_ pellet. Red: Bmpip_2_SnBr_4_ pellet. e,f) Reproduced with permission.^[^ ^129^ ^]^ Copyright 2019, American Chemical Society. g) Schematic diagram of a simplified device for gas sensing application. h) Digital photos of (C_9_NH_20_)_2_MnBr4 upon exposure to six kinds of organic vapors for 10 min under 365 nm UV excitation. g,h) Reproduced with permission.^[^ ^131^ ^]^ Copyright 2019, American Chemical Society.

It is generally believed that LDMHPs have higher PCEs, faster decay and larger stokes shifts compared with 3D ones.^[^ ^122^ ^]^ These advantages demonstrate the potential of LDMHPs for large‐area and low‐cost scintillator devices in the field of medical imaging, nondestructive detection, space exploration, etc. For example, Birowosuto et al.^[^ ^123^ ^]^ reported three X‐ray scintillator characteristics of 3D MAPbI_3_ and MAPbBr_3_ and 2D (EDBE)PbCl_4_ hybrid perovskite crystals. Comparing to 3D perovskites, 2D (EDBE)PbCl_4_ has less thermal quenching due to the large exciton binding energy so that moderate light yield of 9000 photons MeV^−1^ can be obtained even at room temperature (**Figure** **19**a). Moreover, Masanori et al.^[^ ^124^ ^]^ researched 2D organic–inorganic hybrid perovskite‐type scintillator single crystal of 5 × 6 × 1 mm^3^ of (PEA)_2_PbBr_4_ (PEA^+^ = C_6_H_5_(CH_2_)_2_NH_3_^+^). Under 662 keV gamma‐ray excitation, the crystal shows a substantial light yield of 10 000 photons MeV^−1^, with a main decay time component of 9.4 ns. Subsequently, they further optimized the crystal and investigated scintillation properties of (PEA)_2_PbBr_4_ under gamma‐ray and X‐ray irradiations. They fabricated 17 × 23 × 4 mm^3^ bulk sample, which shows a remarkably high light yield of 14 000 photons MeV^−1^ and very fast decay time of 11 ns under gamma‐rays.^[^ ^125^ ^]^

In addition, Xie et al.^[^ ^126^ ^]^ demonstrated Li‐dopant (PEA)_2_PbBr_4_ crystal and explored the application of Li‐(PEA)_2_PbBr_4_ scintillator in alpha particle detection, X‐ray imaging and discrimination between alpha particle and gamma‐ray. It is worth mentioning that they obtained first satisfactory X‐ray imaging pictures of a ubiquitous safety pin using Li‐(PEA)_2_PbBr_4_ film (Figure 19b,c). However, the toxicity of lead in these lead‐based halide perovskites may restrict its potential commercial applications. Thereby, Cao and his colleagues^[^ ^127^ ^]^ developed a lead‐free 2D layered (C_8_H_17_NH_3_)_2_SnBr_4_ perovskite scintillators, which not only exhibit a high PCE of 98% and a large Stokes shift of 246 nm but also provide nontoxicity, good PL intensity, and stability under X‐ray illumination. The results make the novel perovskite scintillators suitable for X‐ray imaging applications (Figure 19d). And Hardhienata et al.^[^ ^128^ ^]^ studied the optical and scintillation properties of manganese‐based 2D organic–inorganic hybrid perovskite crystals X_2_MnCl_4_ (X = PEA, PPA). Both of them show promising PL properties, reasonable decay time (about 3–4 µs) and small bandgaps (about 2 eV), making them applicable for scintillator in X‐ray imaging application. Viktoriia et al.^[^ ^129^ ^]^ reported isostructural 0D halide complexes of Bmpip_2_SnBr_4_ and Bmpip_2_PbBr_4_ (Bmpip = 1‐butyl‐1‐methylpiperidinium cation) that can exhibit potent X‐ray fluorophores being comparable to that of a commercial inorganic NaI:T1 X‐ray scintillator (Figure 19e,f).

At the same time, LDMHPs have also shown potential in transducer field due to their adjustable structural phase transition and fluorescence emissions. For example, Zhou and his colleague^[^ ^130^ ^]^ reported a novel 0D all‐inorganic perovskite single crystal Cs_2_InBr_5_·H_2_O for sensitive water detection. And Li et al.^[^ ^131^ ^]^ have carefully researched the temperature‐dependent phase transition and photoluminescence properties of 0D organic–inorganic hybrid metal halide (C_9_NH_20_)_2_MnBr_4_. Based on this compound, they further developed a fluorescent sensor for Acetone. By simplified gas‐sensitive detecting device with (C_9_NH_20_)_2_MnBr_4_, rapid fluorescence quenching shows when the organic solvent is acetone, which cannot be found when applying other organic solvents (Figure 19g,h).

## 4 Prospects and Outlook

As a new perovskite‐type photovoltaic material system, LDMHPs exhibit excellent stability to water, heat and light, and are expected to solve the instability problem of traditional 3D perovskites, which plays a crucial role for the final industrialization of perovskite materials. By adjusting the dimensions and crystal structures, LDMHPs are expected to provide more opportunity for various optoelectronic devices. However, the LDMHP are still a new type material which has not been fully developed yet, and there are still some problems to be solved

a) How to decrease environmental pollution. Although the stability of organic–inorganic hybrid perovskites has been greatly improved, the potential toxicity problem of Pb^2+^ has not been solved. At present, some people try to use other metal elements to replace or partially replace Pb to prepare lead‐free or lead‐less perovskite solar cells, however, the PCE is not ideal. Public health concerns about lead toxicity will promote the development of lead‐free LDMHPs.
b) Currently, LDMHPs with inherent white emission have many attractive advantages, but their PLQEs are still too low when used in WLEDs. Moreover, there is a lack of systematic researches and understanding of the design, synthesis, and luminescence process mechanism of these LDMHPs. In addition, 1D and 0D perovskite materials still need a lot of work to explore especially for the application to LED devices.
c) Although more and more LDMHPs‐based optoelectronic devices show multifunctional performance and long‐term stability, the properties of intrinsic materials and the composite dynamic process need further research. At the same time, an in‐depth understanding of the structure‐performance relationship is very necessary for the continued development of LDMHPs.

## Conflict of Interest

The authors declare no conflict of interest.

## Author Contributions

Y.H. and S.Y. contributed equally to this work. All the authors contibuted to the discussion of the content, writing and editing of manuscript prior to submission.
